# Anomopterellidae Restored, with Two New Genera and Its Phylogeny in Evanioidea (Hymenoptera)

**DOI:** 10.1371/journal.pone.0082587

**Published:** 2013-12-10

**Authors:** Longfeng Li, Alexandr P. Rasnitsyn, Chungkun Shih, Dong Ren

**Affiliations:** 1 College of Life Sciences, Capital Normal University, 105 Xisanhuanbeilu, Haidian District, Beijing, China; 2 Paleontological Institute, Russian Academy of Sciences, Moscow, Russia; 3 Department of Palaeontology, Natural History Museum, London, United Kingdom; Chinese Academy of Sciences, China

## Abstract

**Background:**

Anomopterellidae was originally classified as a family within the Evanioidea, and later lowered to a subfamily, Anomopterellinae, of Praeaulacidae. Up to date, only Rasnitsyn 1975, with four species, was assigned to Anomopterellinae. Due to their special wing venation and their metasomal attachment similar to those known in Evanioidea, the systematic position of Anomopterellinae in Evanioidea has been in contention.

**Principal Findings:**

Here we report a new fossil genus *Synaphopterella* gen. nov. and six species from the Middle Jurassic of China and transfer *Anomopterella stenocera* Rasnitsyn, 1975, from Upper Jurassic of Kazakhstan, to *Choristopterella* gen. nov. We place these three genera in the restored family Anomopterellidae and provide a key to known genera and species.

**Conclusions/Significance:**

Based on new fossil specimens and phylogenetic analyses, Praeaulacidae has the most basal position in Evanioidea and it is justifiable to restore Anomopterellidae Rasnitsyn, 1975 as a full family. Comparing the size of all described anomopterellids from China, Mongolia and Kazakhstan, we conclude that the species from China have larger bodies and forewings. Diversity of the Praeaulacidae and Anomopterellidae in the late Middle Jurassic of Daohugou suggests that Evanioidea appeared at least before the late Middle Jurassic.

## Introduction

Praeaulacidae, an extinct family of wasp, was proposed as the ancestral group of the whole Evanioidea [1,2]. It comprises four subfamilies: Praeaulacinae, Anomopterellinae, Cretocleistogastrinae and Nevaniinae [3]. Anomopterellidae was originally classified as a family within the Evanioidea [4], and later lowered to a subfamily of Praeaulacidae [5]. Up to date, only Rasnitsyn 1975, with four species, was assigned to Anomopterellinae. These four species are *A. mirabilis* Rasnitsyn, 1975 and *A. stenocera* Rasnitsyn, 1975 from the Late Jurassic of Kazakhstan [4]; *A. huangi* Zhang & Rasnitsyn, 2008 from the Middle Jurassic of China [2]; and *A. gobi* Rasnitsyn, 2008 from the Late Jurassic of Mongolia [6]. Mesonotum transversely ridged is an important diagnostic character in Anomopterellinae, and Praeaulacinae from Daohugou possessed the same transversely ridged mesonotum, a character indicative of parasitic habits on xylophagous insect larvae [5,7]; that is why Zhang and Rasnitsyn [2] supposed that Anomopterellinae had the same feeding habit.

Recently, we collected 12 specimens (including 7 with part and counterpart) from the Middle Jurassic of Daohugou in China. Based on these specimens, *Synaphopterella patula* Li, Rasnitsyn, Shih & Ren, gen. et sp. nov. is erected, and five new species, *A. brachystelis* sp. nov., *A. coalita* sp. nov., *A. ampla* sp. nov., *A. divergens* sp. nov., and *A. ovalis* sp. nov., are assigned to *Anomopterella* Rasnitsyn, 1975. Additionally, *Anomopterella stenocera* Rasnitsyn, 1975, is transferred to *Choristopterella* gen. nov., and the diagnosis of *Anomopterella* is modified. The new material shows some characters are variable in this group, though they are stable in other families of Evanioidea. These are the medial length of the pronotum and presence of the medial mesoscutal suture, which are, respectively, long and present in Praeaulacidae, and short and absent in Evaniidae s.l. (including Cretevaniidae) and Gasteruptiidae s.l. (including Aulacidae and Baissidae). Together with striking apomorphies of Anomopterellidae in their fore and hind wing venation (see diagnoses below), these features justify the restoration of this group as a full family and not a subfamily of Praeaulacidae.

Many fossil hymenopterons have been found in Daohugou Village, Inner Mongolia, China [8-12]. The fossil-bearing beds contain abundant fossils of plants, insects and other animals [13-18]. The section at Daohugou Village is composed of grey tuffaceous sandstone and sandy mudstone [19,20]. According to the accurate Ar-Ar and SHRIMP U-Pb dating, the age of Daohugou fossil-bearing beds is Jiulongshan Formation, the late Middle Jurassic (Bathonian-Callovian boundary, 165 million years ago [Mya]) [21].

## Materials and Methods

### Materials

All 12 fossil specimens studied are housed in the Key Lab of Insect Evolution and Environmental Changes, College of Life Sciences, Capital Normal University (CNU) in Beijing, China (CNUB; Dong Ren, Curator). No specific permits were required for the described field studies.

### Methods

All specimens were examined using a Leica MZ12.5 dissecting microscope and illustrated with the aid of a camera lucida attached to the microscope. The figures were drawn using CorelDraw 12.0 and Adobe Photoshop CS5. Wing venation terminology is basically from Rasnitsyn (1969) [22].

Venation nomenclature: Rs, radial sector; M, media; Cu, cubitus; 1r‑rs, the first radial crossvein; 2r‑rs, the second radial crossvein; cu-a, the first cubito-anal crossvein; 2cu‑a, the second anal crossvein; 1m-cu, the first mediocubital crossvein; 2m-cu, the second mediocubital crossvein; 2r‑m, the second radiomedial crossvein; 3r‑m, the third radiomedial crossvein.

### Phylogenetic analysis

Phylogenetic analyses including 20 morphological characters, of which 12 are wing characters and 8 are body characters, were conducted. The character selection is based on the phylogenetic analysis of the Evanioidea [23] and the suborder phylogeny of Vespina [24]. The characters and their states are defined in Table 1. The cladogram is rooted using the following three outgroup families: Karatavitidae Rasnitsyn, 1963; Ephialtitidae Handlirsch, 1906; Kuafuidae Rasnitsyn & Zhang 2010. The outgroup selection is based on the suborder phylogeny of Vespina [24], considering that the Karatavitidae is basal in respect to Ephaltitidae, Ephaltitidae is basal in respect to Evanioidea, and the position of Kuafuidae between Ephaltitidae and the higher Apocrita. The ingroups consist of five families described up to date, including two extinct families: Praeaulacidae Rasnitsyn, 1972 and Anomopterellidae restored herein, and three extant famies: Evaniidae Latreille, 1802, Gasteruptiidae Kirby, 1837 (including Kotujellitinae Rasnitsyn, 1990) and Aulacidae Shuckard, 1841 (including Baissinae Rasnitsyn, 1975). The character matrix of outgroups and Evanioidea is provided in Table 2.

**Table 1:** Definition of characters and their states.

1. Head: (0) ordinary, not modified; (1) long (=high, i.e. from ocelli to mouth), flattened posteriorly.
2. Number of antennal segments: (0) ≥14; (1) 13; (2) 12
3. Medial mesoscutal suture: (0) present; (1) absent
4. Notauli: (0) either join transscutal suture or effaced posteriorly or completely lost; (1) meet posteriorly before reaching the transscutal suture.
5. Pronotum: (0) long; (1) short
6. Propleurae: (0) moderately short, head more or less adpressed to prothorax; (1) elongate, forming long neck.
7. Wing fixation apparatus (cenchri + riugh area within a loop of 2A vein): (0) present in outgroup Karatavitidae (except genus *Karatavites*); (1) lost in all Apocrita (except rudimentary in few Ephialtitidae).
8. Forewing with first abscissa of RS: (0) directed distal, nearly parallel M+Cu; (1) directed subvertical, forming distinct angle with Rs+M; (2) directed posterobasal, forming acute angle with Rs+M.
9. Forewing lr-rs: (0) complete; (1) not reaching pterostigma, often rudimentary or lost.
10. Forewing 2r-m: (0) present, or rudimentary on Rs and M; (1) entirely lost
11. Forewing 3r-m: (0) present (at least rudimentary on RS and M); (1) entirely lost
12. Forewing 2A: (0) present; (1) absent (except rarely present in Praeaulacidae)
13. Forewing a_1_-a_2_: (0) present; (1) absent
14. Forewing 2m-cu: (0) present (at least rudimentary on M and Cu); (1) entirely lost
15. Forewing with marginal cell: (0) moderately or particularly wide (or incompletely closed); (1) wide tranglular
16. Hind wing cell r: (0) only enclosed (in Karatavitidae, Ephialtitidae, Kuafuidae and Praeaulacidae); (1) open (in the remaining families including Ephialtitidae and Praeaulacidae which are polymorphic).
17. Hind wing jugal lobe (posterobasal wing ares): (0) delimited by a fold and tucking under the wing at rest; (1) secondarily not delimited (lost).
18. Hind wing m-cu: (0) present; (1) lost
19. Metasomal attachment to propodeum: (0) metasoma attach high on propodeum; (1) metasoma attach low on propodeum, near hind coxae.
20. Ovipositor: (0) surpassing abdominal (metasomal) apex variable in Ephialtitidae, Praeaulacidae and possibly in some other families considered; (1) surpassing abdominal (metasomal) apex is correct for Anomopterellidae.

**Table 2:** Character matrix of 20 characters for the eight taxa included in this study.

**Taxa/ character**	**1**	**2**	**3**	**4**	**5**	**6**	**7**	**8**	**9**	**10**	**11**	**12**	**13**	**14**	**15**	**16**	**17**	**18**	**19**	**20**
**Karatavitidae**	0	0	0	0	0,1	0	0	0	0	0	0	0	0	0	0	0	0	0	0	0
**Ephialtitidae**	0	0	0	0	0,1	0	1	1,2	1	0	0,1	1	0	0	0	0,1	1	1	0	0
**Kuafuidae**	0	0	0	?	0,1	0	1	1,2	1	0	0	1	0	0	0	0	1	1	1	0
**Evaniidae**	1	2	1	0	1	0	1	1,2	1	0,1	0,1	1	1	1	0	1	0	1	0	0
**Gasteruptiidae**	0	0,1	1	1	1	0,1	1	2	1	1	0,1	1	1	1	0	1	1	1	0	0
**Aulacidae**	0	1	1	0	1	0,1	1	2	1	0,1	0	1	1	0	0	1	1	1	0	0
**Praeaulacidae**	0	0	0	0	0	0	1	2	1	0,1	0,1	1	1	0,1	0	0,1	1	1	0	0
**Anomopterellidae**	0	0	0,1	0	0,1	0	1	2	1	1	0	1	1	0	1	1	?	1	0	1

?, state unknown

Phylogenetic analyses were undertaken in NONA 2.0 (Goloboff 1997) [25], with the options of “hold 10,000; mult 1,000” and in WinClada version 1.00.08 interface (Nixon 2002) [26]. Character codings were set up by using Nexus Data Editor 0.5.0 (Roderic 2001) [27] with all characters unordered and of equal weight.

### Nomenclatural Acts

The electronic edition of this article conforms to the requirements of the amended International Code of Zoological Nomenclature, and hence the new names contained herein are available under the Code from the electronic edition of this article. This published work and the nomenclatural acts it contains have been registered in Zoobank, the online registration system for the ICZN. The Zoobank LSIDs (Life Science Identifiers) can be resolved and the associated information viewed through any standard web browser by appending the LSID to the prefix "Http://zoobank.org". The ISID for this publication is: urn:lsid:zoobank.org:pub:A3D7C3CC-3619-4BCB-BD68-1B9FCFB9677F. The electronic edition of this work was published in a journal with an ISSN, and has been archived and is available from the following digital repositories: PubMed Central and LOCKSS.

## Results

### Systematic Palaeontology

Order Hymenoptera Linnaeus, 1758
Suborder Apocrita Gerstaecker, 1867
Superfamily Evanioidea Latreille, 1802
Anomopterellidae Rasnitsyn, 1975

#### Type genus

*Anomopterella* Rasnitsyn, 1975

#### Other genera included

*Synaphopterella* Li, Rasnitsyn, Shih & Ren, gen. nov. and *Choristopterella* Li, Rasnitsyn, Shih & Ren, gen. nov.

#### Diagnosis

Forewing with 8 enclosed cells; 1-RS much longer than 1-M; 2r‑m lost; 2r‑rs meeting pterostigma near its apex; 3r‑m and 2m-cu present; cell 2mcu as wide (high) as, or narrower than cell 2+3 rm; 3-Cu subequal to, or shorter than 1m-cu and much shorter than 2cu‑a; 2A and a1-a2 absent. Hind wing with C present; Rs and M fused for an interval; free terminal of Cu absent. Pronotum variable in respect to its medial length; mesonotum variable in respect to presence or absence of medial suture. First metasomal segment narrow basally. Ovipositor short.

#### Remarks

Unlike Anomopterellidae, all other Evanioidea have 3r‑m distant from 2m-cu (or 2m-cu lost), 3-Cu longer, 2cu‑a shorter, cell 2mcu always wider (higher) even if open, and hind wing with RS and M never fused. Praeaulacidae always has pronotum long medially and mesonotum with medial suture; in contrast, Evaniidae and Gasteruptiidae always have pronotum short medially and mesonotum without medial suture. Also, Praeaulacidae has ovipositor short only in combination with two-segmented petiole which never occurs in Anomopterellidae. Evaniidae additionally differs in having 1-Rs subequal to, or shorter than 1-M, and 2m-cu lost.

#### Key to known genera and species of the family Anomopterellidae

**1.** Forewing with Rs+M aligned with M+Cu (1-M lost), 3r-m and 2m-cu coincide, 1-Cu subvertical and about as long as 1cu-a. Pronotum and propodeum both short.

  ............... .....*Synaphopterella* gen. nov. (one species, *S. patula* sp. nov.)

- Forewing with Rs+M not aligned with M+Cu, 1M present; 3r-m and 2m-cu not coincident; 1cu-a interstitial or less postfurcal (much longer than 1-Cu).   ....................2

**2.** Forewing with Rs+M is separated from 1m-cu by a considerable distance. Rs starting near pterostigma, angular at 2r-rs. Antenna thin, at least as long as forewing. Propodeum very short, metasoma attaching to its vertical surface. Forewing length < 2.5 mm. ....................

..........................*Choristopterella* gen. nov. (one species, *C. stenocera* (Rasnitsyn, 1975))

- Forewing with Rs+M reaching 1m-cu. Antenna thick. Propodeum longer. Forewing length > 2.5 mm.................... *Anomopterella* Rasnitsyn, 1975. ...................................3

**3.** Forewing with cu-a interstitial. ................................4

- Forewing with cu-a postfurcal. .....................................6

**4.** Forewing with Rs origin close to pterostigma, Rs very short between 2r-rs and 3r-m. Forewing length 3.5 mm.........................................................................*A. gobi* Rasnitsyn, 2008

- Forewing with Rs starting far from pterostigma, Rs long between 2r-rs and 3r-m, 1r-rs absent, forewing length > 5 mm........................................ .5

**5.** First metasomal segment elongate triangular (length/width ratio between 1.2 and 1.6).......................................*A. coalita* sp. nov.

- First metasomal segment wide triangular (length / width ratio 1.1)....................*A. ampla* sp. nov.

**6. (3)** RS starting from pterostigma for a distance comparable with length of 1m-cu. Ovipositor long, far surpassing metasomal apex. First metasomal segment wide triangular (length / width ratio 1.0). Forewing length 2.6 mm....................*A. mirabilis* Rasnitsyn, 1975.

- RS starting far from pterostigma. Ovipositor short, weakly extending beyond metasomal apex. Forewing length above 4 mm. ...............................7

**7.** Rudimentary 1r-rs about as long as 1m-cu. First metasomal segment particularly narrow with distinct petiole (length / width ratio 1.7). Forewing length about 5 mm...................................*A. brachystelis* sp. nov.

- Rudimentary 1r-rs much shorter or lost. First metasomal segment elongate triangular.........................8

**8.** Rudimentary 1r-rs absent. Forewing length about 4.5 mm...........*A. divergens* sp. nov.

Rudimentary 1r-rs dot-like small. Forewing length about 5 mm.........................9

**9.** First metasomal segment short (length / width ratio 0.9). Legs much longer and narrow............................*A. huangi* Zhang & Rasnitsyn, 2008

- First metasomal segment elongate triangular (length / width ratio 1.4). Legs shorter and slightly wide............................*A. ovalis* sp. nov.

*Choristopterella* Li, Rasnitsyn, Shih & Ren, gen. nov.

urn:lsid:zoobank.org:act:37D9CBD3-FCFE-4482-A90C-6FA4C121E497

#### Type species

*Anomopterella stenocera* Rasnitsyn, 1975

#### Diagnosis

Mesosoma short, high. Pronotum short medially. Propodeum high, strongly convex and quite short, with posterior margin subvertical. Forewing with Rs starting near pterostigma, angular at 2r‑rs. Vein 2r‑rs meeting pterostigma near apex; Rs+M is separated from 1m-cu by a considerable distance; cu-a postfurcal. Antenna very thin, at least as long as forewing.

#### Remarks

*C. stenocera* (Rasnitsyn, 1975) was previously classified as a species of the genus *Anomopterella*. The new material described below, shows that the species differs sufficiently from the species of *Anomopterella* to be separated as a genus of its own.

#### Etymology

The generic name is a combination of Greek “*choristos*” (separate) referring to Rs+M is separated from 1m-cu by a considerable distance, and the generic name *Anomopterella*. Gender: feminine.

*Synaphopterella* Li, Rasnitsyn, Shih & Ren, gen. nov

urn:lsid:zoobank.org:act:594492B4-0F8C-44FC-848B-E3EAFDBAF3E4.

#### Type species.

*Synaphopterella patula* Li, Rasnitsyn, Shih & Ren, sp. nov.

#### Diagnosis.

Antenna moderately thick. Mesosoma short, pronotum and propodeum both short. Forewing with 2r‑rs meeting pterostigma near apex; 1r‑rs rudimentary. Rs+M aligned with M+Cu (1-M lost), 3r‑m and 2m-cu coincide, 1-Cu subvertical and about as long as 1cu‑a.

#### Remarks.

Very specific venation, with RS+M aligned with M+Cu, so that cell 1mcu is shifted below the level of the latter, has an analogy found only in the distantly related, living genus *Gasteruption* Latreille, 1796 (Gasteruptiidae: Gasteruptiinae).

#### Etymology.

The generic name is a combination of Greek “*synaphes*” (contiguous, contacting), referring to the coincident 3r‑m and 2m-cu, and the generic name *Anomopterella*. Gender: feminine.

**Figure 1:**
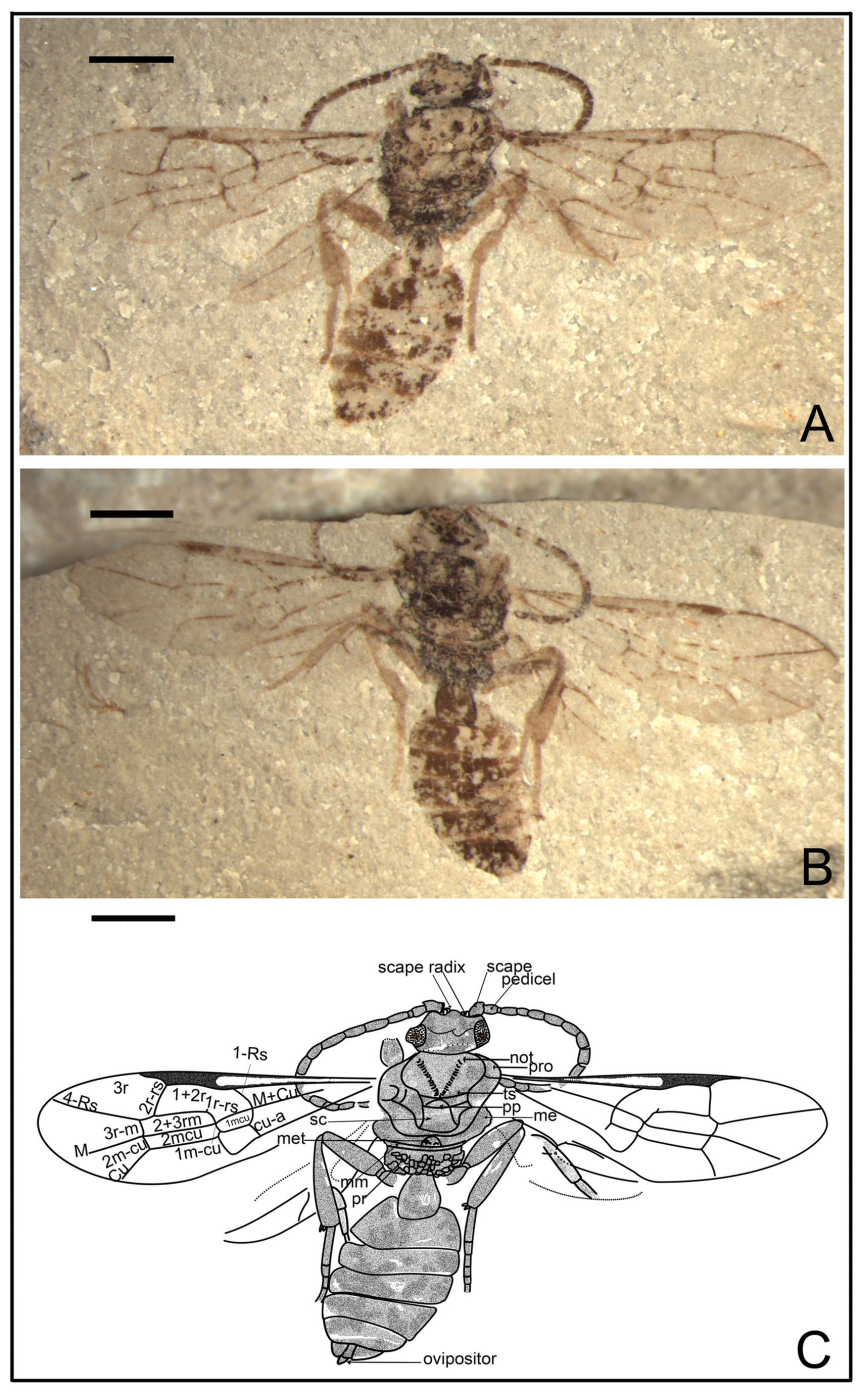
Synaphopterella patula sp. nov. Photographs (A), (B) and line drawing (C) of Holotype CNU-HYM-NN-2012019(P/C); Scale bars: 1 mm. me, metanotum; met, Metascutellum; mm, metapostnotum; not, notaulus; pp, praescutellar pit; pr, propodeum; pro, pronotum; sc, scutellum; ts, transscutal suture.

*Synaphopterella patula* Li, Rasnitsyn, Shih & Ren, sp. nov. (Fig. 1)

urn:lsid:zoobank.org:act:29C58EAA-B6C2-480B-9AEC-630A09D71101.

#### Holotype.

CNU-HYM-NN-2012019(P/C), a well-preserved specimen with complete body and forewings.

#### Diagnosis.

As for genus, and see the key above.

#### Description.

Holotype CNU-HYM-NN-2012019(P/C), size small, length of body 4.7 mm, forewing length 4.0 mm. Head transversely ovoid, width 1.5 times length. Antenna with 14 segments as preserved; scape slightly longer than pedicel; almost as long as flagellomeres; flagellomeres of subequal length and width, some 2.5-3 times as long as wide. Mesosoma wider than head in dorsal aspect; pronotum very short, probably covered by mesonotum; mesonotum with transscutal sutures and notauli distinct, and notauli nearly V-shaped, with medial suture not apparent; prescutellar pit and Metascutellum semi-circular, scutellum nearly trapezoid; metanotum transversely wider than metapostnotum, Metascutellum apparently with a pair of posterior pits (as in Ichneumonidae: Ichneumoninae), metapostnotum very short. Forewing venation with Rs origin at a distance from pterostigma, 1r‑rs rudimentary, like a very short stub on Rs; 2r‑rs meeting pterostigma near apex, 2r‑rs vertical and 1.5 times as long as maximal width of 2+3 rm; cell 1+2rs narrower than 3r, 3r quite broad, triangular; cell 2+3 rm twice as long as, and slightly wider than 1mcu, and as long as, and distinctly wider than 2mcu; 2r‑rs meeting Rs basad of 3r-m for length of 3r‑m, Rs+M reaching 1m-cu, cu-a interstitial. Hind leg 3.99 mm: femur, 1.20 mm; tibia, 1.78 mm; tarsus, 1.01 mm. Hind tibia with two small apical spurs minutely serrated. First metasomal segment comparatively thin basally, gradually broadened apically, 1.3 times as long as wide (length 0.60 mm, maximum width 0.48 mm). Ovipositor short, very slightly extending beyond metasomal apex.

#### Locality and horizon.

Collected near Daohugou Village, Shantou Township, Ningcheng County, Inner Mongolia, China, the Middle Jurassic.

#### Etymology.

From the Latin “*patula*” meaning “expanded”, referring to the well-preserved and complete forewings.

*Anomopterella* Rasnitsyn, 1975

#### Type species.

*Anomopterella mirabilis* Rasnitsyn, 1975

#### Additional species included.

*A. huangi* Zhang & Rasnitsyn, 2008; *A. gobi* Rasnitsyn, 2008; *A. brachystelis* sp. nov., *A. coalita* sp. nov., *A. ampla* sp. nov., *A. divergens* sp. nov., and *A. ovalis* sp. nov.

#### Emended diagnosis.

Antenna moderately or quite thick. Mesosoma short, high. Propodeum high, strongly convex or quite short, with posterior margin subvertical. Forewing venation with 1r‑rs absent or rudimentary like a stub, M+Cu not aligned with RS+M, 1-M present, 2r‑rs and 2m-cu basad of 3r‑m. Vein cu-a interstitial or slightly postfurcal. Metasoma broadest beyond its mid-length, with first segment gradually thickening distally and longer than any other segments. Ovipositor short and only slightly extending beyond metasomal apex (possibly more extending in type species), with sheaths shorter than basal sclerite.

**Figure 2:**
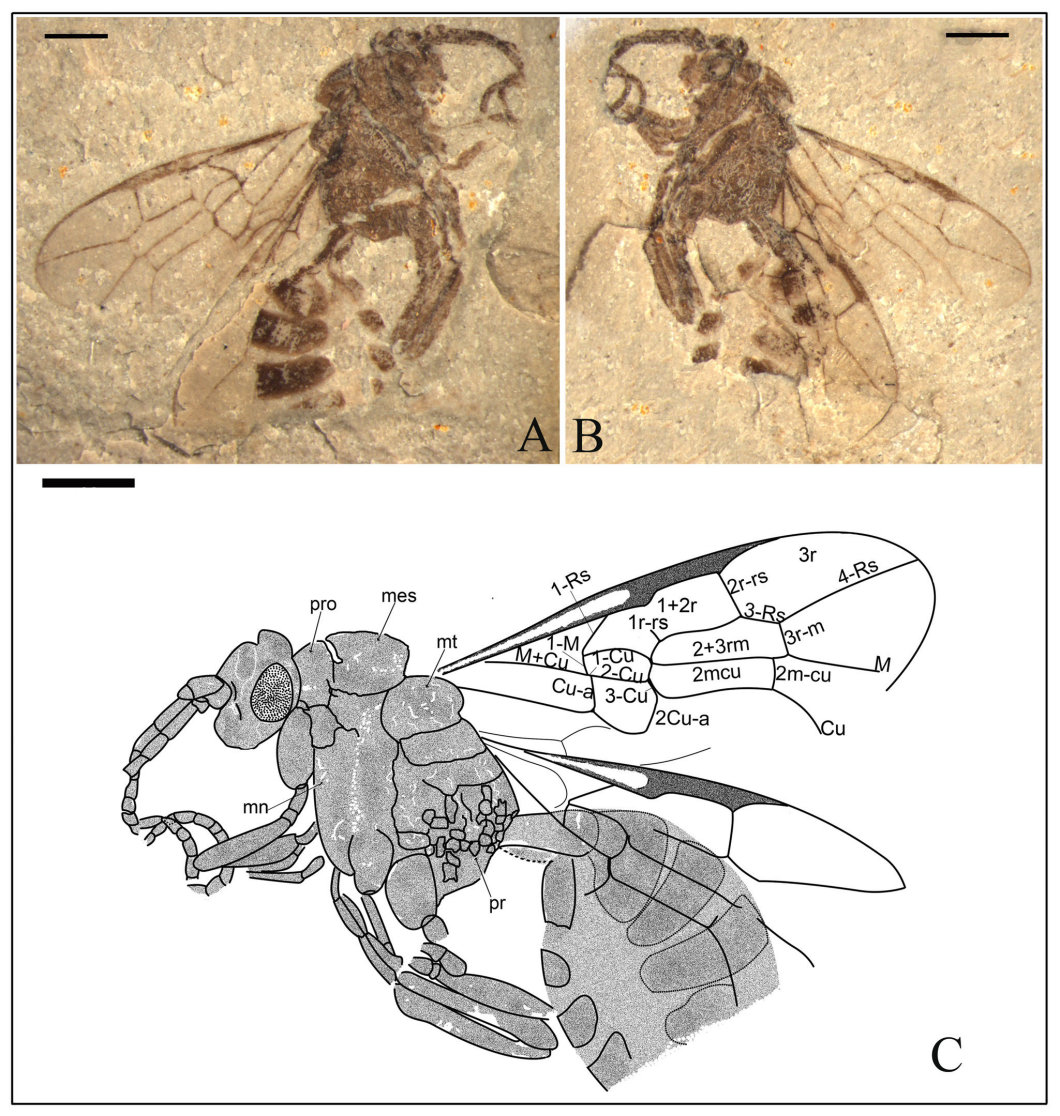
*Anomopterella brachystelis* sp. nov. Photographs (A), (B) and line drawing (C) of Holotype CNU-HYM-NN-2012020(P/C); Scale bars: 1 mm. mes, mesonotum; mn, mesopleuron; pr, propodeum; pro, pronotum.

**Figure 3:**
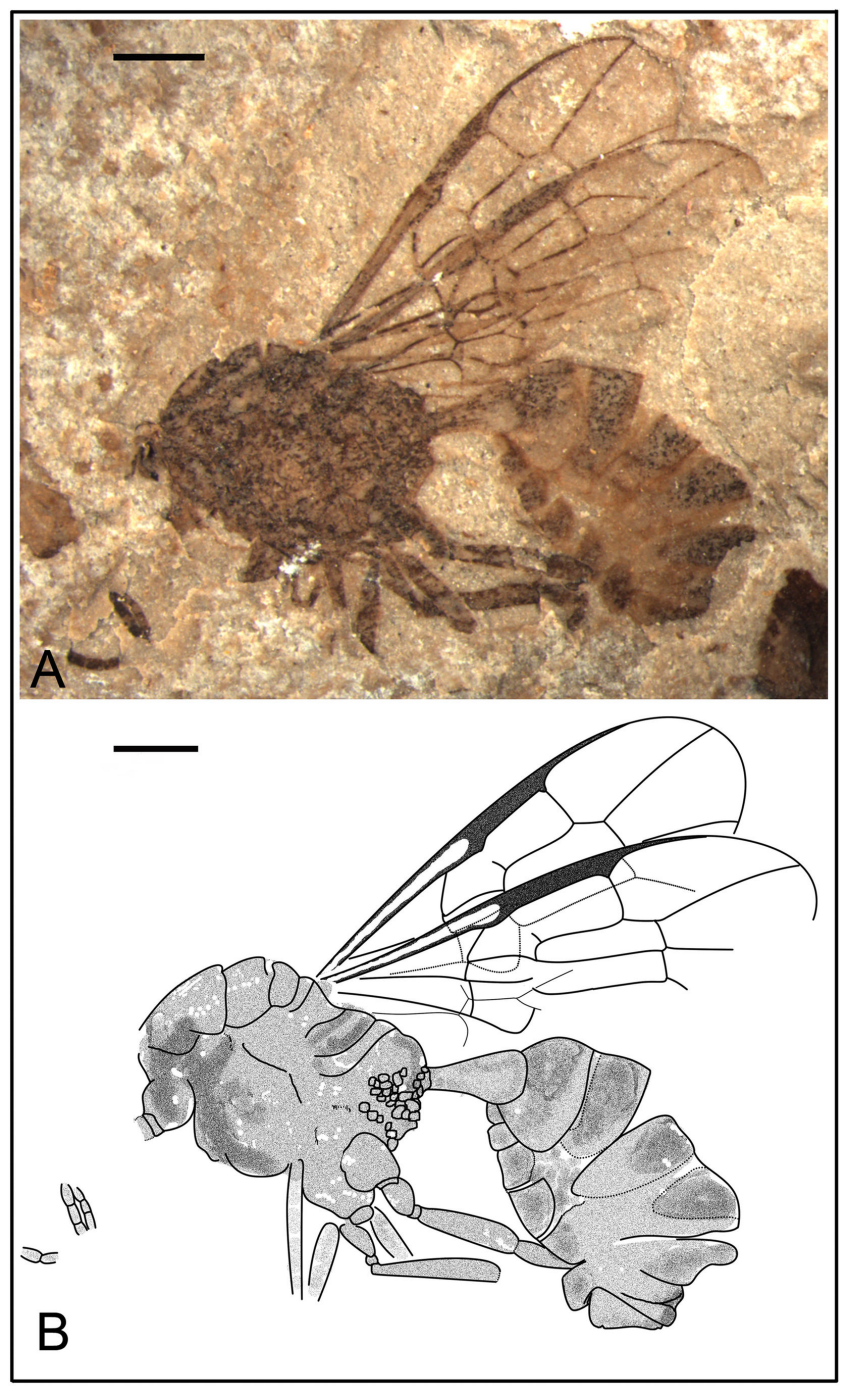
*Anomopterella brachystelis* sp. nov. Photograph (A), and line drawing (B) of Paratype CNU-HYM-NN-2012025; Scale bars: 1 mm.

*Anomopterella brachystelis* Li, Rasnitsyn, Shih & Ren, sp. nov. (Figs. 2, 3)

urn:lsid:zoobank.org:act:E737FE5D-0B5B-4A63-B185-F6AF15C38A77.

#### Holotype.

CNU-HYM-NN-2012020(P/C), a well-preserved specimen with nearly complete body and wings. Paratype CNU-HYM-NN-2012025, an incomplete specimen, head not preserved, two wings partially overlapping.

#### Diagnosis.

Forewing with Rs origin at a distance from pterostigma, 1r‑rs rudimentary like a stub, Rs long between 2r‑rs and 3r‑m, 2r‑rs and 2m-cu basad of 3r‑m, cu-a postfurcal. First metasomal segment particularly narrow with a distinct short petiole.

#### Description.

Holotype CNU-HYM-NN-2012020(P/C) (Figure 2), length of body 6.4 mm, forewing length 4.9 mm. Head almost as wide as mesosoma. Antenna with 15 segments as preserved, inserted below mid height of eyes, with scape wider than pedicel; pedicel very short, transverse; first flagellomere segment longer than following segments; eye large and ovoid. Mesonotum nearly 2 times as long as pronotum in side aspect; mesoscutellum nearly as long and wide as mesonotum, both of them round as squashed ball; mesopleuron 2 times as high as wide, propodeum broad with posterior margin subvertical. Forewing with 1-Rs about 2 times as long as 1-M; 1r‑rs rudimentary like a stub; Rs long between 2r‑rs and 3r‑m, 2r‑rs and 2m-cu basad of 3r‑m; cell 1+2rs slightly narrower than 3r, 3r quite broad, triangular; cell 1mcu and 2+3 rm in contact, and 2+3 rm twice as long as and slightly wider than 1mcu; cu-a postfurcal. Legs incomplete, forelegs thinner and shorter than hindlegs. First metasomal segment comparatively thin basally, gradually broadened apically with a short petiole, 1.7 times as long as wide (length 0.83 mm, maximum width 0.48 mm).

Paratype CNU-HYM-NN-2012025 (Figure 3), wing venation similar to holotype except for some length of veins and body structure. Forewing length 4.95 mm. First metasomal segment comparatively thin basally, gradually broadened apically with a short petiole, 1.7 times as long as wide (length 0.97 mm, maximum width 0.56 mm).

#### Locality and horizon.

Collected near Daohugou Village, Shantou Township, Ningcheng County, Inner Mongolia, China, the Middle Jurassic.

#### Etymology.

From the Latin “*brachystelis*” meaning “short of handle”, referring to the first metasomal segment particularly narrow with a short petiole.

**Figure 4:**
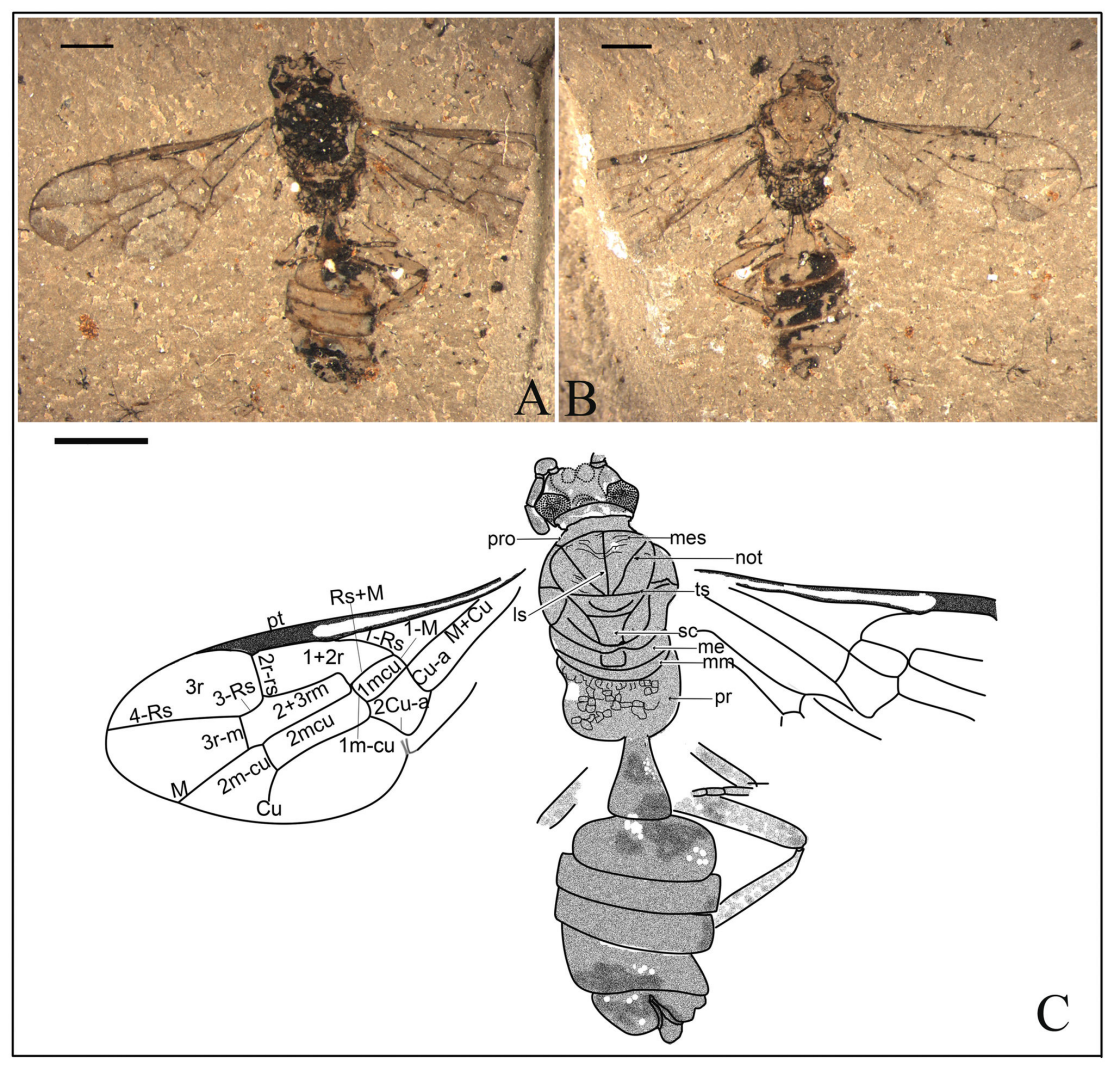
*Anomopterella coalita* sp. nov. Photographs (A), (B) and line drawing (C) of Holotype CNU-HYM-NN-2012030(P/C); Scale bars: 1 mm. ls, medial suture; me, metanotum; mes, mesonotum; mm, metapostnotum; not, notaulus; pr, propodeum; pro, pronotum; sc, scutellum; ts, transscutal suture.

**Figure 5:**
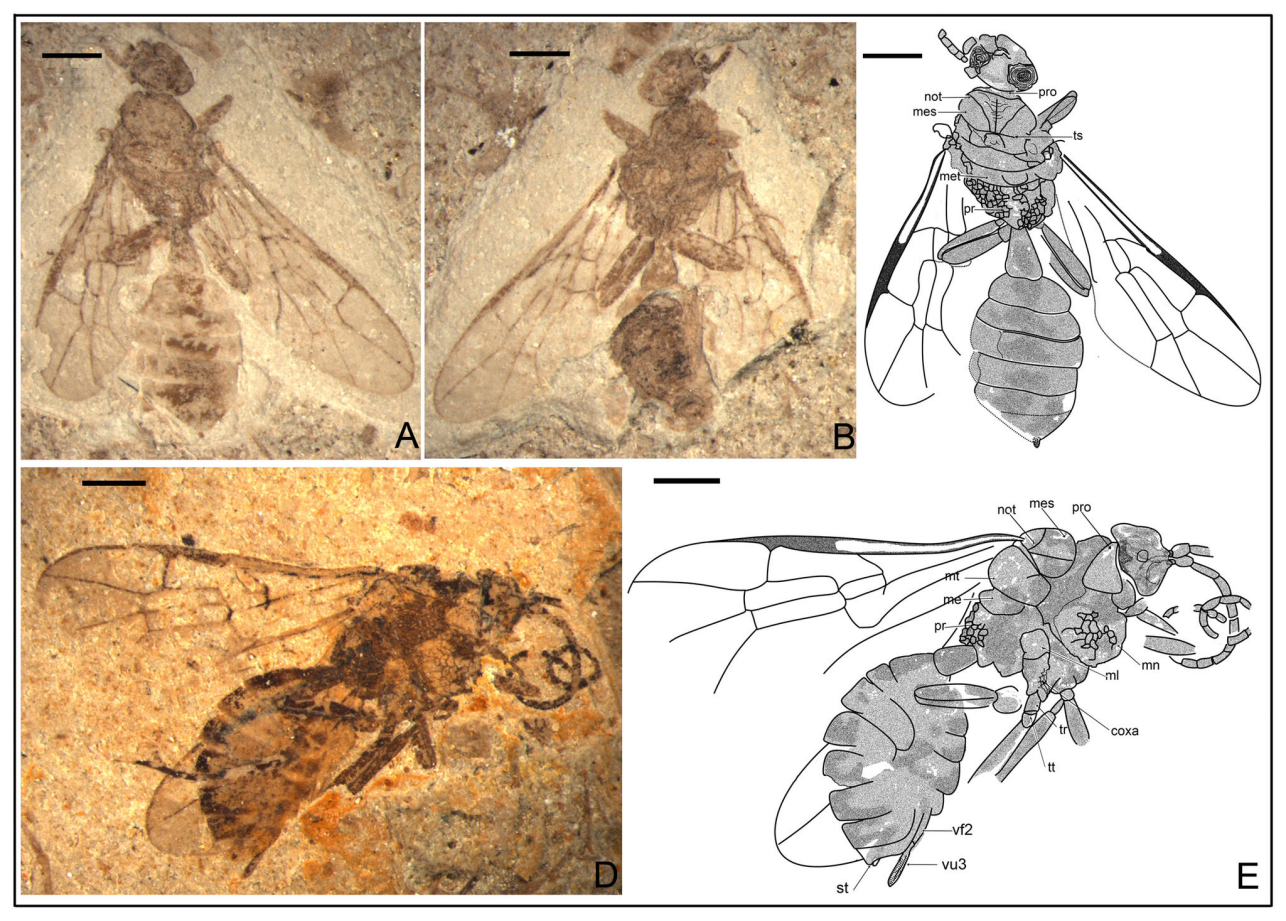
*Anomopterella coalita* sp. nov. Photographs (A), (B) and line drawings (C) of Paratype CNU-HYM-NN-2012023 (P/C); (D), (E) Paratype CNU-HYM-NN-2012028; Scale bars: 1 mm. me, metanotum; mes, mesonotum; met, Metascutellum; ml, metapleuron; mm, metapostnotum; mn, mesopleuron; mt, mesoscutellum; not, notaulus; pr, propodeum; pro, pronotum; st, stylus; tr, trochanter; ts, transscutal suture; tt, trochantellus; vf2, valvifer; vu3, valvula3.

*Anomopterella coalita* Li, Rasnitsyn, Shih & Ren, sp. nov. (Figs. 4, 5)

urn:lsid:zoobank.org:act:83DC4D48-D355-4D6F-BBDD-028A3D578687.

#### Holotype.

CNU-HYM-NN-2012030(P/C), a well-preserved specimen without complete antenna and legs. Paratypes CNU-HYM-NN-2012023(P/C), with asymmetrical forewings, right forewing longer and narrower than left forewing, indicating possible deformation of the matrix during the fossilization process; CNU-HYM-NN-2012028; and CNU-HYM-NN-2012029.

#### Diagnosis.

Forewing with Rs origin at a distance from pterostigma; 1r‑rs absent; Rs long between 2r‑rs and 3r‑m, 2r‑rs and 2m-cu basad of 3r‑m; cu-a interstitial. The first metasomal segment elongate triangular.

#### Description.

Holotype CNU-HYM-NN-2012030(P/C) (Figure 4), length of body 6.4 mm, forewing length 5.0 mm. Head normal in size, with large eyes. Antenna with scape distinctly wider than pedicel. Pronotum short, probably covered by mesonotum; mesonotum transversely ridged with V-shape notauli, median and transscutal suture; scutellum trapezoid, metanoum nearly as wide as metapostnotum, both short; propodeum broad and coarsely areolate. Forewing with Rs origin at a distance from pterostigma; 1r‑rs absent; 1-Rs about 3 times as long as 1-M; Rs long between 2r‑rs and 3r‑m, 2r‑rs and 2m-cu basad of 3r‑m; cell 1+2rs slightly narrower and shorter than 3r, 3r quite broad, triangular; cell 2+3 rm nearly as wide as 2mcu; cu-a interstitial. Hind wing with first abscissa of Rs and M at an obtuse angle. First metasomal segment comparatively thin basally, gradually broadened apically and slightly longer than wide, elongate triangular, 1.2 times as long as wide (length 0.90 mm, maximum width 0.74 mm).

Paratype CNU-HYM-NN-2012023(P/C) (Figure 5 A, B, C), body length 6.8 mm, forewing length 5.0 mm. Head transversely ovoid with large eyes. Mesosoma broad and ovoid in dorsal aspect; pronotum very short, possibly covered by mesonotum; mesonotum transversely ovoid with distinct V-shaped notauli and transscutal suture; propodeum broad and with fine and dense reticulation. Forewing with Rs origin at a distance from pterostigma; 1r‑rs absent; 1-Rs about 3 times as long as 1-M; 2r‑rs vertical and 1.2 times as long as maximal width of 2+3 rm; 3r‑m and 2m-cu present, cell 2mcu nearly as long and wide as 2+3 rm; cu-a interstitial. First metasomal segment elongate triangular, 1.4 times as long as wide (length 0.81 mm, maximum width 0.59 mm). Ovipositor slightly exposed.

Paratype CNU-HYM-NN-2012028 (Figure 5 D, E), head normal in size, wider than pronotum; antenna with 10 segments preserved, scape distinctly thicker than pedicel. Mesosoma broad and round in profile aspect, pronotum nearly triangular-shape; Mesonotum comparatively long, metapleuron wide, reaching metacoxal base; propodeum broad with posterior margin subvertical. Forewing length 5.4 mm, forewing with 1-Rs about 3 times as long as 1-M; 2r‑rs vertical and 1.3 times as long as maximal width of 2+3 rm; 3r‑m and 2m-cu present, cell 2mcu as long and wide as 2+3 rm; cu-a interstitial. Legs incomplete, hind femur distinctly thicker and longer than fore femur; trochanter thin basally, becoming broad and longer than wide; femur elongate with trochantellus distinct; tibia nearly as long as femur. First metasomal segment elongate triangular, 1.5 times as long as wide (length 0.60 mm, maximum width 0.40 mm). Ovipositor short, stylus present like teardrop-shaped, valvifer 2 slightly shorter and narrower than valvula 3, length of valvifer 2 about 0.57 mm, valvula 3 about 0.52mm long.

#### Locality and horizon.

Collected near Daohugou Village, Shantou Township, Ningcheng County, Inner Mongolia, China, the Middle Jurassic.

#### Etymology.

From the Latin “*coalita*” meaning “united”, referring to forewing venation cu-a interstitial.

**Figure 6:**
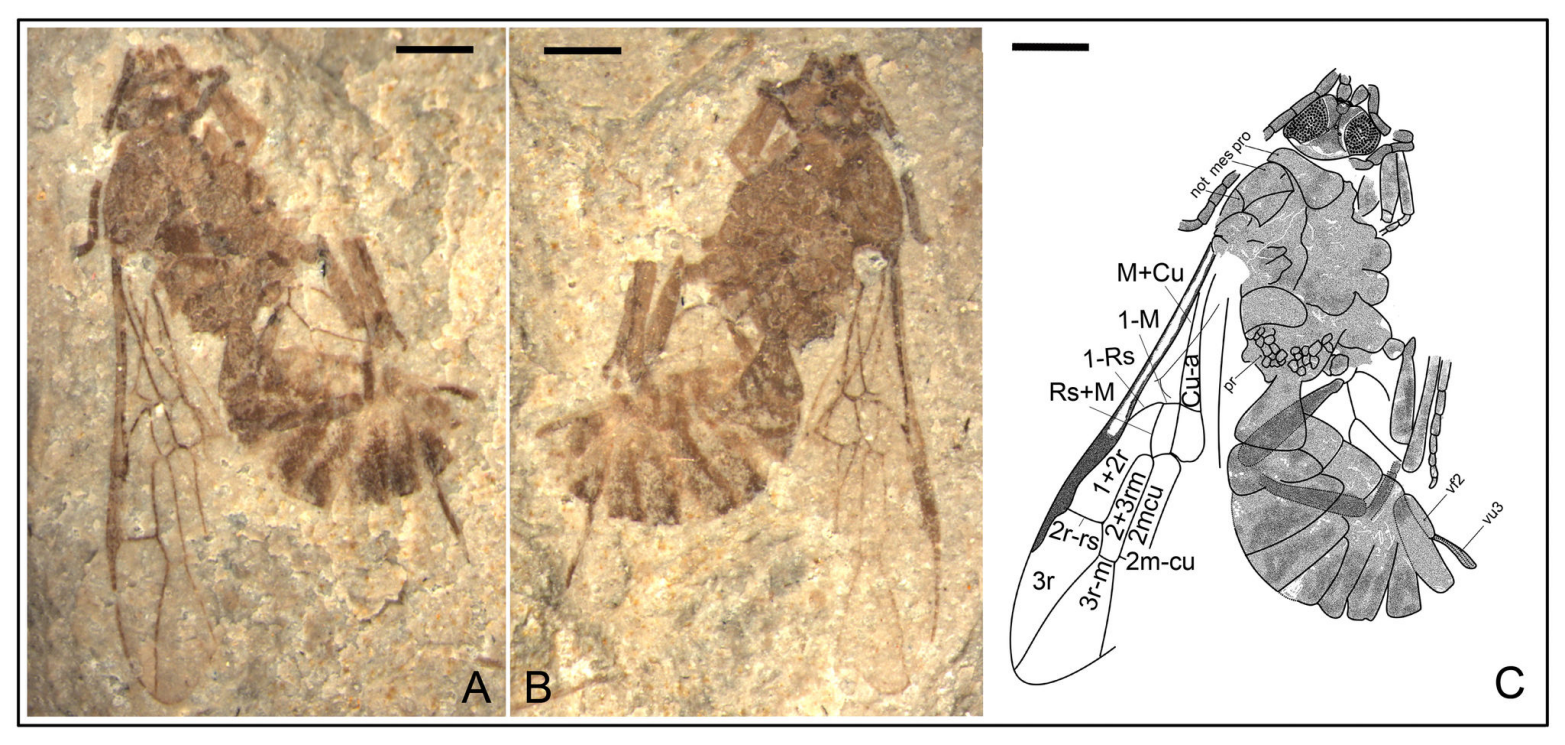
*Anomopterella ampla* sp. nov. Photographs (A), (B) and line drawing (C) of Holotype CNU-HYM-NN-2012024 (P/C); Scale bars: 1 mm. mes, mesonotum; not, notaulus; pr, propodeum; pro, pronotum; vf2, valvifer; vu3, valvula3.

*Anomopterella ampla* Li, Rasnitsyn, Shih & Ren, sp. nov. (Fig. 6)

urn:lsid:zoobank.org:act:30EA7E86-867F-4A22-849A-C748B7B016AD.

#### Holotype.

CNU-HYM-NN-2012024 (P/C), an incomplete female wasp with antennae, legs and wings partly preserved, mesosoma and metasoma nearly complete.

#### Diagnosis.

Forewing with Rs origin at a distance from pterostigma, 1r‑rs absent, Rs long between 2r‑rs and 3r‑m, 2r‑rs and 2m-cu basad of 3r‑m, cu-a interstitial; First metasomal segment wide triangular.

#### Description.

Holotype CNU-HYM-NN-2012024(P/C), body length 7.3 mm, forewing length 5.7 mm. Head transversely ovoid, with large eyes. Antenna insertion slightly above eye midheight, with 10 segments as preserved. Scape distinctly wider than pedicel, the basal flagellomere segments longer than following segments. Pronotum transversely spoon-shaped, mesonotum round with distinct notauli; propodeum broad with fine and dense reticulation. Forewing with Rs origin at a distance from pterostigma; 1r‑rs absent; 1-Rs about 2 times as long as 1-M. 2r‑rs vertical and 1.5 times as long as maximal width of cell 2+3 rm. Rs long between 2r‑rs and 3r‑m, 2r‑rs and 2m-cu basad of 3r‑m; cu-a interstitial. Legs incomplete, hind femur and tibia thicker and longer than fore femur and tibia. First metasomal segment wide triangular, 1.1 times as long as wide (length 0.86 mm, maximum width 0.82 mm). Ovipositor short, valvifer 2 slightly wider than valvula 3, length of valvifer 2 about 0.60 mm, valvula 3 about 0.58 mm long.

#### Locality and horizon.

Collected near Daohugou Village, Shantou Township, Ningcheng County, Inner Mongolia, China, the Middle Jurassic.

#### Etymology.

From the Latin “*ampla*” meaning “wide”, referring to the first metasomal segment wide triangular.

**Figure 7:**
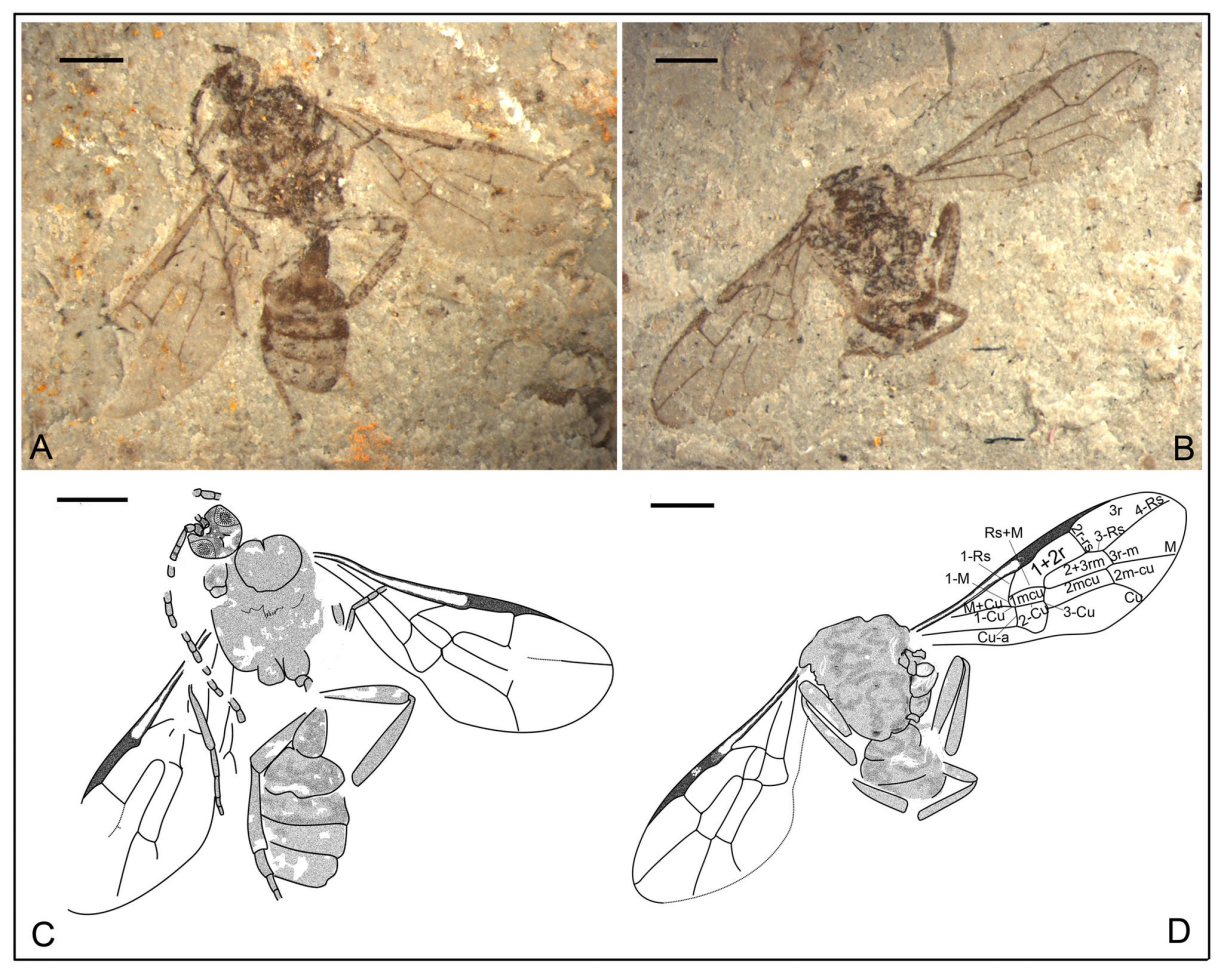
*Anomopterella divergens* sp. nov. Photographs and line drawings of *A. divergens* sp. nov. (A), (C) Holotype CNU-HYM-NN-2012027; (B), (D) Paratype CNU-HYM-NN-2012026; Scale bars: 1 mm.

*Anomopterella divergens* Li, Rasnitsyn, Shih & Ren, sp. nov. (Fig. 7)

urn:lsid:zoobank.org:act:C86BFCD7-F9DB-49C1-A7C6-7F0CF227FE85.

#### Holotype.

CNU-HYM-NN-2012027, in dorsal aspect. An incomplete wasp of unknown sex with antenna and wings partially preserved, mesosoma and metasoma poorly preserved. Paratype CNU-HYM-NN-2012026, with head and parts of metasoma missing, the first metasomal segment not preserved.

#### Diagnosis.

Forewing with Rs origin at a distance from pterostigma, 1r‑rs absent, Rs+M reaching 1m-cu, cu-a postfurcal. The first metasomal segment elongate triangular.

#### Description.

Holotype CNU-HYM-NN-2012027 (Figure 7 A, C), body length 5.1 mm, forewing length 4.6 mm. Head normal in size, rounded with compound eye. Antenna insertion slightly above the midpoint of eyes, with 16 segments preserved, scape and pedicel short, the first flagellomere longer than following segments. Forewing with 1r‑rs absent; vein 2m-cu present; cu-a postfurcal. Legs incomplete, hind femur shorter and wider than tibia (femur 1.36 mm long, and 0.24 mm at greatest width; tibia 1.49 mm long, and 0.19 mm at greatest width). The first metasomal segment elongate triangular, 1.6 times as long as wide (length 0.73 mm, maximum width 0.45 mm).

Paratype CNU-HYM-NN-2012026 (Figure 7 B, D), forewing length 4.45 mm, forewings complete and outspread. Forewing with Rs origin at a distance from pterostigma; 1r‑rs absent; cell 1+2rs slightly shorter and narrower than 3r; 3r quite broad, nearly triangular; cells 1mcu and 2+3 rm in contact, and 2+3 rm twice as long as and slightly wider than 1mcu; Rs long between 2r‑rs and 3r‑m, 2r‑rs and 2m-cu basad of 3r‑m, 2mcu slightly narrow than 2+3 rm; 1-M and 1-Rs forming nearly a straight line; cu-a postfurcal.

#### Locality and horizon.

Collected near Daohugou Village, Shantou Township, Ningcheng County, Inner Mongolia, China, the Middle Jurassic.

#### Etymology.

From the Latin “*divergens*” meaning “divaricate or separate”, referring to forewing venation cu-a postfurcal.

**Figure 8:**
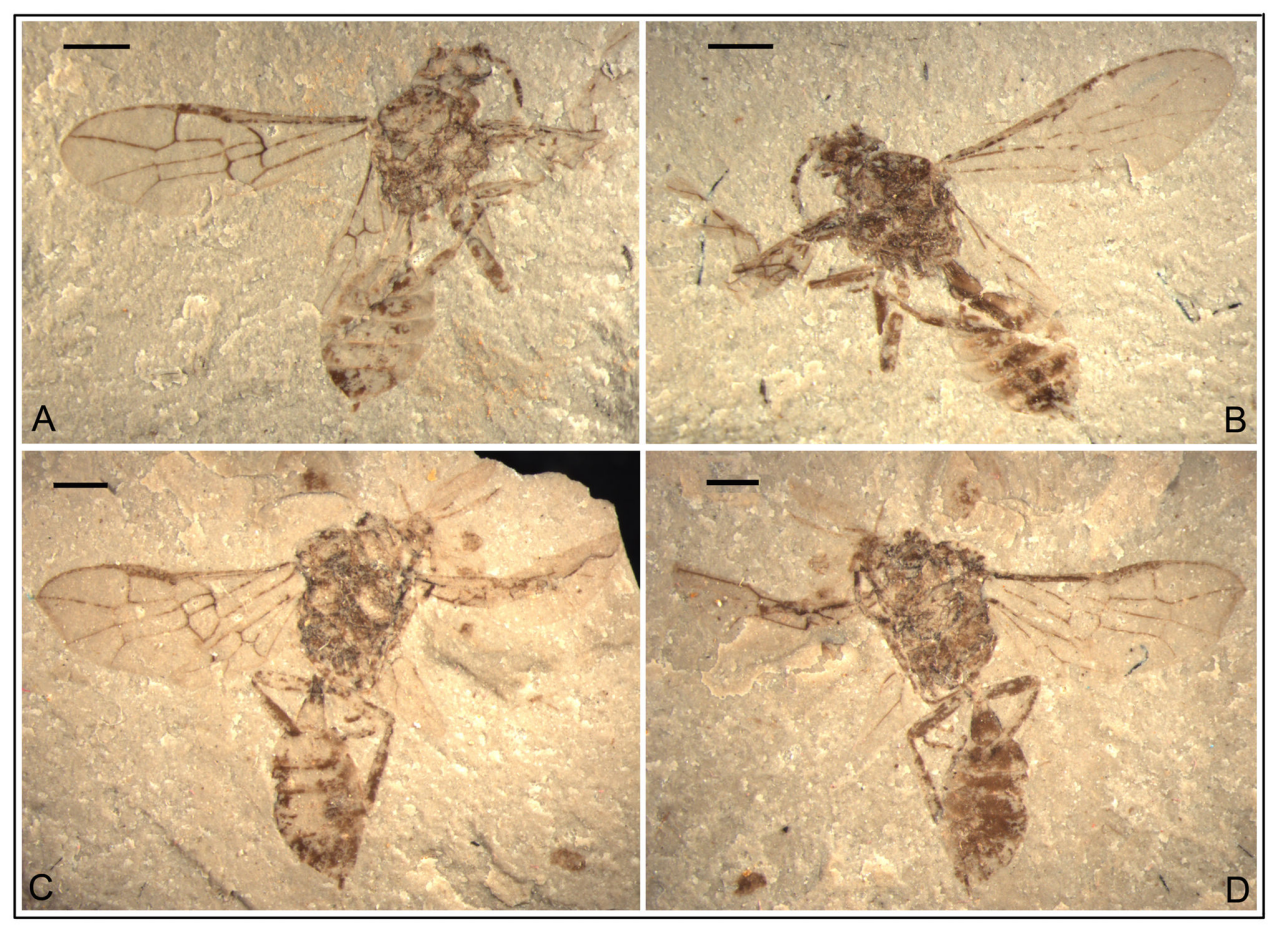
*Anomopterella ovalis* sp. nov. Photographs (A), (B) of Holotype CNU-HYM-NN-2012021(P/C); (C), (D) Paratype CNU-HYM-NN-2012022 (P/C); Scale bars: 1 mm.

**Figure 9:**
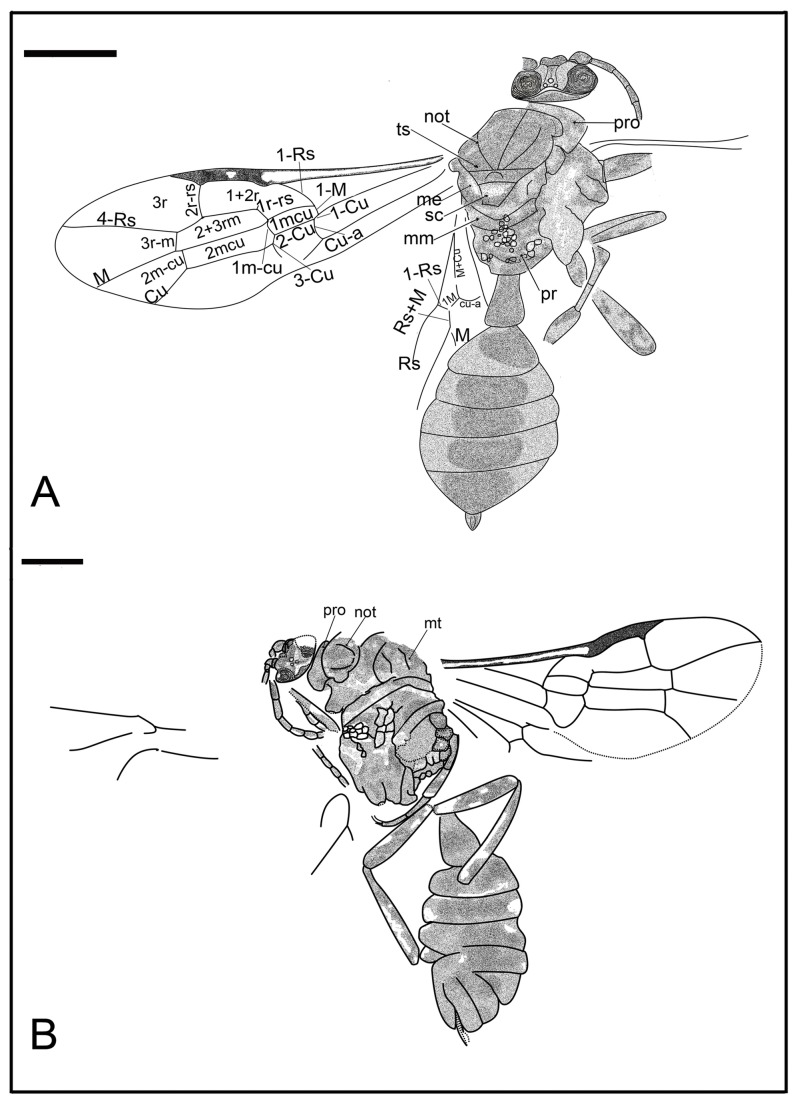
*Anomopterella ovalis* sp. nov. Line drawings (A) Holotype CNU-HYM-NN-2012021(P/C); (B) Paratype CNU-HYM-NN-2012022 (P/C); Scale bars: 1 mm. me, metanotum; mm, metapostnotum; mt, mesoscutellum; not, notaulus; pr, propodeum; pro, pronotum; sc, scutellum; ts, transscutal suture.

*Anomopterella ovalis* Li, Rasnitsyn, Shih & Ren, sp. nov. (Figs. 8, 9)

urn:lsid:zoobank.org:act:A323D81F-9CE0-4F02-8B03-C6D28F59CCA3.

#### Holotype.

CNU-HYM-NN-2012021(P/C) with well preserved forewings, mesosoma and metasoma. Paratype CNU-HYM-NN-2012022(P/C), an incomplete female wasp, in profile aspect, with antennae, wings and partly preserved legs. Shape of forewing indicates that forewing and its associated matrix were distorted during the fossilization process.

#### Diagnosis.

Forewing with Rs origin at a distance from pterostigma; 1r‑rs rudimentary, like a very short stub on Rs; Rs long between 2r‑rs and 3r‑m, 2r‑rs and 2m-cu basad of 3r‑m; cu-a postfurcal, and first metasomal segment elongate triangular.

#### Description.

Holotype CNU-HYM-NN-2012021(P/C) (Figure 8 A, B; Figure 9 A), length of body 5.8 mm, forewing length 4.7 mm. Head slightly wider than long, ocelli present and compound eyes relatively large. Antenna with 5 segments preserved, scape wider than pedicel. Mesosoma broad, and ovoid in dorsal aspect; pronotum very short, partly covered by mesonotum; mesonotum with transscutal, median suture and notauli distinct; scutellum trapezoid, metanotum nearly as wide as metapostnotum, both short, propodeum broad. Forewing with costal area beyond Rs origin slightly wider than pterostigma; 1-Rs about 4 times as long as 1-M and longer than its distance to pterostigma; 1r‑rs rudimentary, like a very short stub on Rs; cell 1+2r slightly narrower than 3r; Rs long between 2r‑rs and 3r‑m, 2r‑rs and 2m-cu basad of 3r‑m; cu-a postfurcal. Metasoma with first segment comparatively thin basally, gradually broadened apically, elongate triangular, 1.4 times as long as wide (length 0.64 mm, maximum width 0.45 mm), remaining part of the metasoma oval in dorsal aspect, ovipositor short.

Paratype CNU-HYM-NN-2012022(P/C) (Figure 8 C, D; Figure 9 B), head small, transversely ovoid. Antennae with 10 flagellomeres preserved, several subbasal ones comparatively thick and 2.5 times as long as wide, distal ones becoming gradually thinner. Forewing length 5.0 mm, forewing with f1‑Rs twice as long as 1-M, 2r‑rs meeting pterostigma near apex; 1r‑rs rudimentary like a very short stub on Rs; veins 3r-m and 2m-cu present; cells 2+3 rm and 2mcu nearly rectangular; cu-a postfurcal. Hind wing with Rs and M meeting at obtuse angle (more than 120°). Legs incomplete, hind femur thicker and shorter than hind tibia (femur: 1.33 mm long, 0.32 mm at greatest width; tibia: 1.69 mm long, 0.23 mm at greatest width). First metasomal segment comparatively thin basally, gradually broadened apically, 1.4 times as long as wide (length 0.83 mm, maximum width 0.61 mm). Ovipositor short.

#### Locality and horizon.

Daohugou Village, Shantou Township, Ningcheng County, Inner Mongolia, China, the Middle Jurassic.

#### Etymology.

From the Latin “*ovalis*” meaning “ovate”, referring to the oval shape of mesosoma and metasoma except for the first segment.

### Phylogeny of the Evanioidea

**Figure 10:**
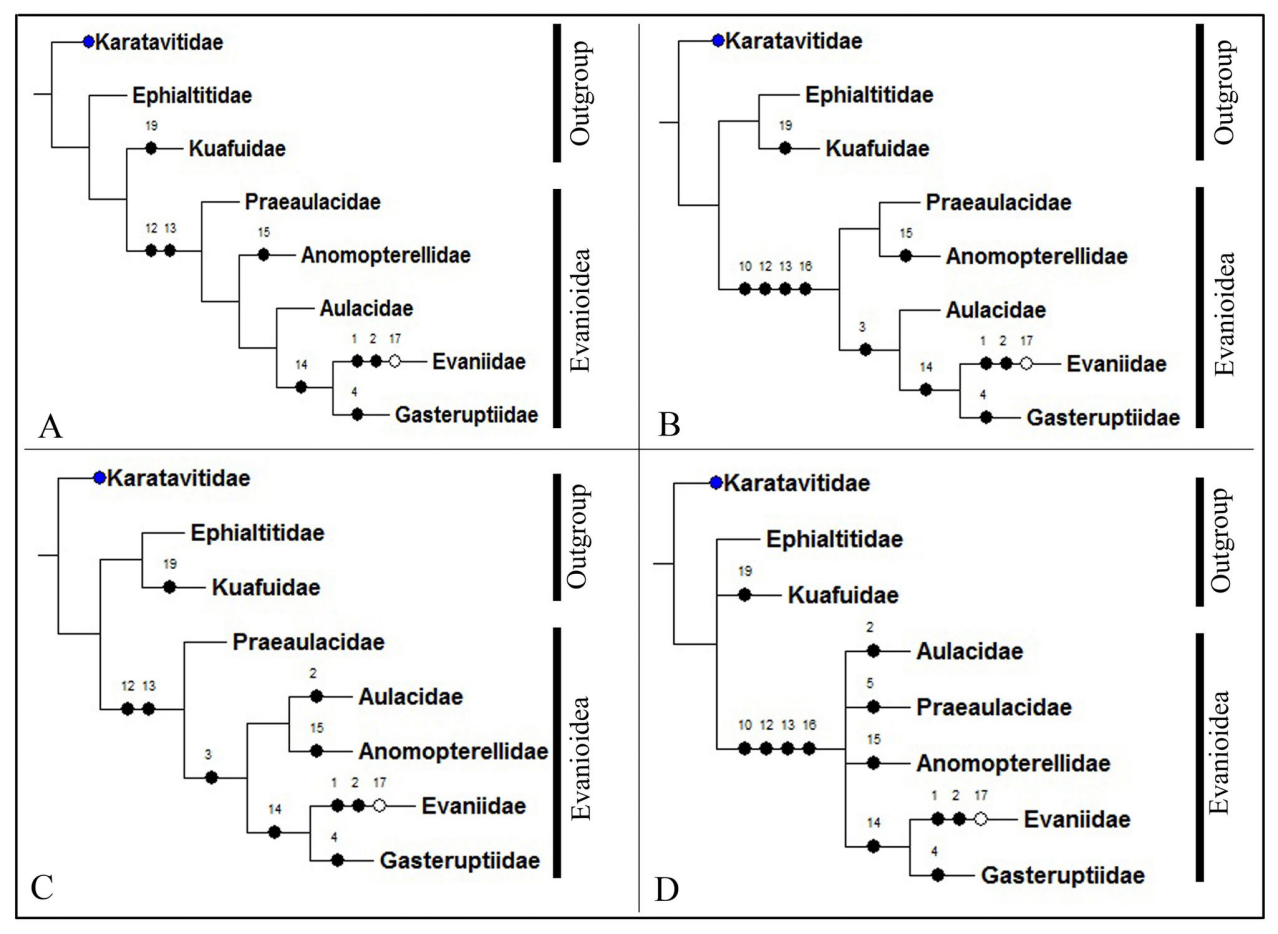
Phylogenetic analysis (results of NONA with three outgroups). A The most parsimonious tree 1; B The most parsimonious tree 2; C The most parsimonious tree 3; D The strict consensus tree. (●) non-homoplasious; (○) homoplasious.

The cladistic heuristic analysis resulted in three equally most parsimonious cladograms (tree length = 19 steps; consistency index = 0.94; retention index = 0.90), as presented in Figure 10 A, B, C. The strict consensus cladogram is shown in Figure 10 D. The major conclusions of our phylogenetic analysis are as follows: Evanioidea is a monophyletic group, which includes five families, forming a large clade supported by four characters: forewing with 2r-m entirely lost except some species of Evaniidae, Aulacidae and Praeaulacidae (char. 10:1); forewing with 2A absent except rarely present in Praeaulacidae (char. 12:1); forewing with a_1_-a_2_ absent (char. 13:1) ; hind wing with cell r open except some species of Praeaulacidae enclosed (char. 16:1). In Evanioidea, Praeaulacidae, at the base of the ingroups, is supported by pronotum long (char. 5:0). Evaniidae and Gasteruptiidae form a sister group supported by forewing with 2m-cu entirely lost (char. 14:1), whereas Aulacidae is assigned as its sister group supported by number of antennal segments is 13 (char. 2:1). Anomopterellidae forms an independent branch because of forewing with marginal cell wide triangular (char. 15:1). Therefore, the phylogenetic results show that Praeaulacidae has the most basal position in Evanioidea and the rest four families are well segregated. Anomopterellidae forming a single branch support the restoration of Anomopterellidae Rasnitsyn, 1975 as a full family, and no longer a subfamily in Praeaulacidae.

## Discussion

**Figure 11:**
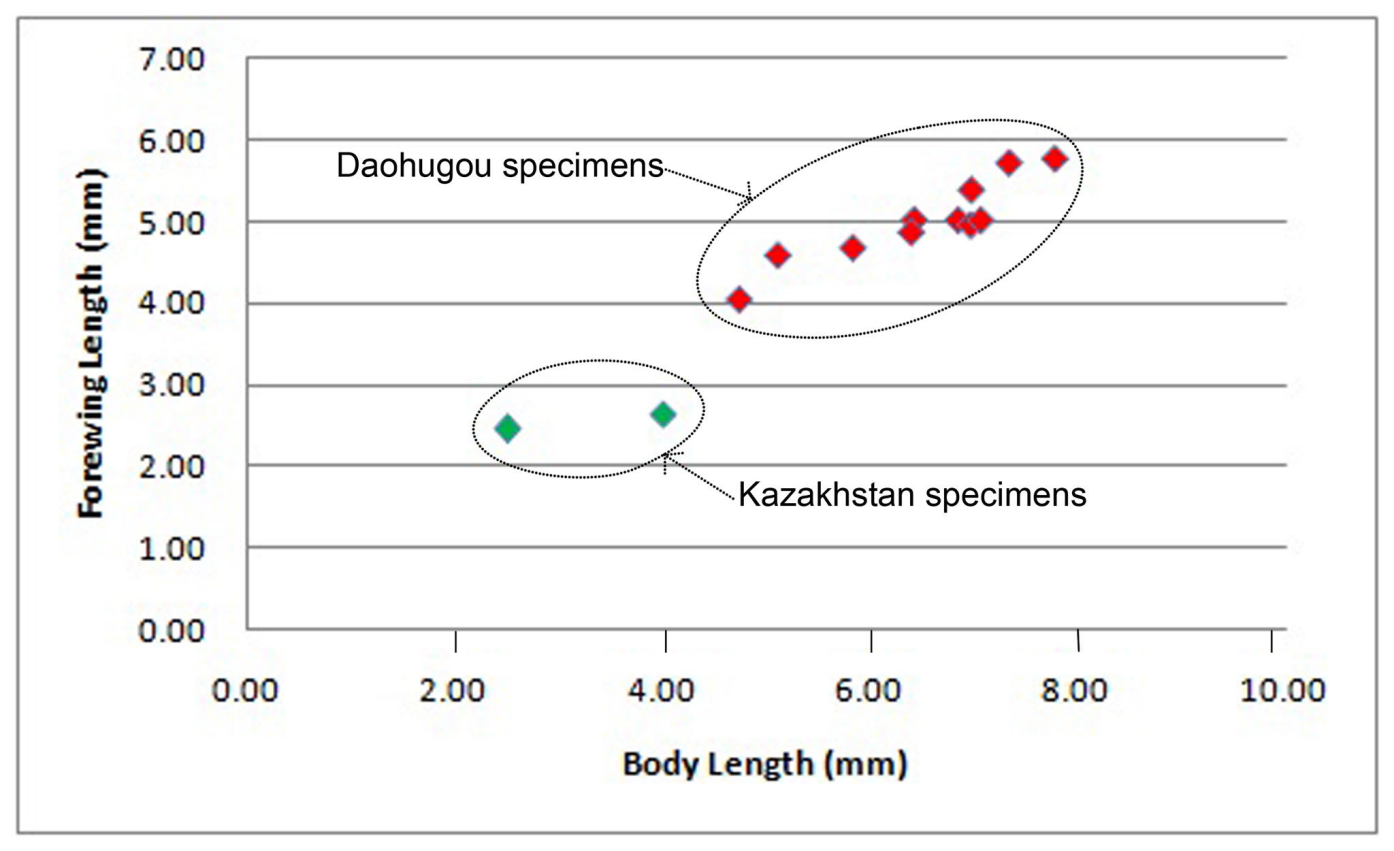
Comparison of body length and forewing length of species of Anomopterellidae.

The lengths of the body and forewing for all known anomopterellids are summarized in Table 3, and forewing lengths vs. body lengths are plotted in Figure 11. The body lengths of Anomopterellidae vary from 2.50 to 7.76 mm, and the forewing lengths from 2.44 and 5.76 mm. In general, the Daohugou specimens are larger than Kazakhstan specimens, with the only known Mongolian isolated forewing being intermediate in length. Larger body size for Daohugou specimens imply warmer climate or more favorable ecosystem in Daohugou than in Kazakhstan. Broader size range for Daohugou specimens suggest that diverse food sources and broad varieties and sizes of parasitic hosts matching with various sizes of anomopterellids had existed in their ecosystems, which is consistent with the proposal by Shih et al., 2010 [28].

**Table 3:** Summary of all species of Anomopterellidae.

**Species Name**		**Specimen ID number**		**Body Length (mm)**	**Forewing length (mm)**	**Vein 1r-rs**	**Vein cu-a**
***S. patula* sp. nov.**		**CNU-HYM-NN-2012019P/C**		4.7	4.0	Present	Interstitial
***Ch. stenocera* (Rasnitsyn, 1975)**		**PIN 2239/2562**		2.5	2.4	Absent	Postfurcal
***A. coalita* sp. nov.**			**CNU-HYM-NN-2012023P/C**	6.4	5.0	Absent	Interstitial
			**CNU-HYM-NN-2012030P/C**	6.8	5.0	Absent	Interstitial
			**CNU-HYM-NN-2012028**	7.0	5.4	Absent	Interstitial
***A. ampla* sp. nov.**			**CNU-HYM-NN-2012024P/C**	7.3	5.7	Absent	Interstitial
***A. brachystelis* sp. nov.**			**CNU-HYM-NN-2012025**	6.4	4.9	Present	Postfurcal
			**CNU-HYM-NN-2012020P/C**	6.9	4.9	Present	Postfurcal
***A. divergens* sp. nov.**			**CNU-HYM-NN-2012026**	5.1	4.6	Absent	Interstitial
			**CNU-HYM-NN-2012027**	not available	4.5	Absent	Interstitial
***A. mirabilis* Rasnitsyn, 1975**			**PIN No 2239/2562**	4.0	2.6	Absent	Postfurcal
***A. gobi* Rasnitsyn, 2008**			**PIN 4270/1549**	not available	3.5	Absent	Interstitial
***A. huangi* Zhang & Rasnitsyn, 2008**			**NND2006/NIGP148228**	not available	5.0	Present	Postfurcal
***A. ovalis* sp. nov.**	**CNU-HYM-NN-2012022P/C**			5.8	4.7	Present	Postfurcal
	**CNU-HYM-NN-2012021P/C**			7.1	5.0	Present	Postfurcal
**A. sp.**	**CNU-HYM-NN-2012029**			7.8	5.8	Absent	Interstitial

In Anomopterellidae, the first segment of the metasoma presents three different shapes: (1) wide triangular, as shown by *A. mirabilis* and *A. ampla* sp. nov. (Figure 6); (2) elongated triangular, as observed in *A. huangi*, *S.* patula sp. nov. (Figure 1), *A. coalita* sp. nov. (Figures 4, 5), *A. divergens* sp. nov. (Figure 7) and *A. ovalis* sp. nov. (Figures 8, 9); (3) particularly narrow with a short petiole, as exhibited in *A. brachystelis* sp. nov. (Figures 2, 3). But all species of Anomopterellidae (except for *A. gobi* of which the metasoma is not preserved), had the articulation of the metasoma and mesosoma arising near the dorsal-most surface of the mesosoma. The metasomal morphology of Anomopterellidae shows characters similar to those known in other Evanioidea.

For *Anomopterella*, two other characters are also considered to be plesiomorphic in hymenoptera: (1) Postfurcal crossvein cu-a in forewing, as shown in *A. mirabilis*, *A. huangi*, *A. brachystelis* sp. nov., *A. divergens* sp. nov., and *A. ovalis* sp. nov. (Figures 2, 3, 7, 8, 9), which is a general feature in sawfly. Therefore, it can be treated as a plesiomorphic character; (2) The presence of crossvein 1r‑rs (although vestigial) in forewings of *A. huangi*, *A. brachystelis* sp. nov. and *A. ovalis* sp. nov. (Figures 2, 3, 8, 9). The crossvein 1r‑rs, which is common in Symphyta but rudimentarily in some Apocrita (e.g. some Ephialtitidae) [4], can be considered as a ground plan trait. These two conditions show that *A. huangi*, *A. brachystelis* sp. nov., and *A. ovalis* sp. nov. possess less derived characters than their congeners, and probably occupy the most basal position of the clade of *Anomopterella*. Furthermore, *Choristopterella* gen. nov. is the most plesiomorphic anomopterellid in respect of RS+M short and not reaching 1m-cu, although, this genus is highly apomorphic in small size and in very short propodeum and comparatively low metasomal attachment.

**Figure 12:**
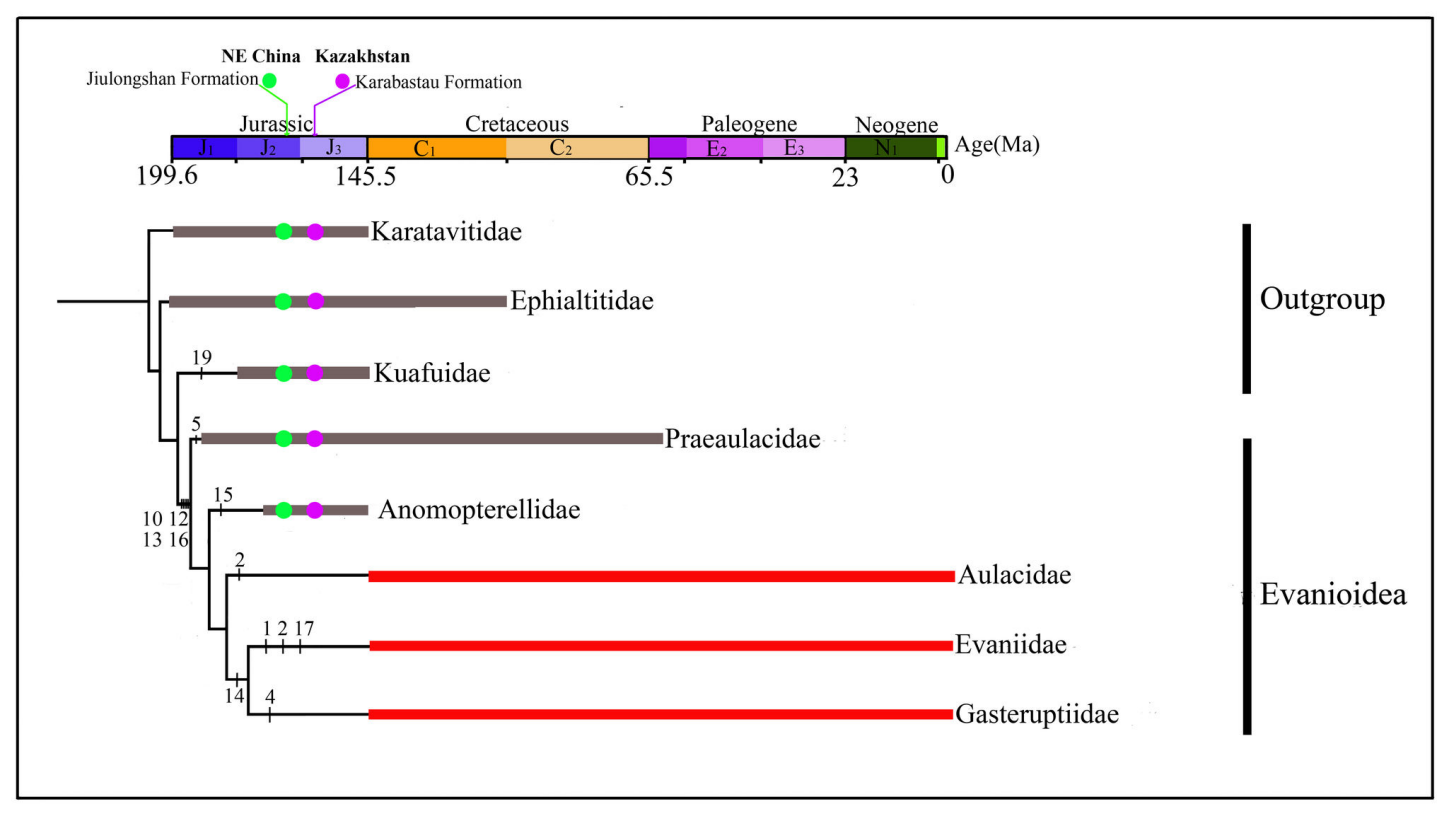
The phylogenetic relationships of 5 families of Evanioidea and 3 outgroups based on morphological characters. Red lines are the known extant taxa of Evanioidea, gray lines are extinct taxa. Color dots show different fossil sites: green dots indicate Middle Jurassic Jiulongshan Formation of China; purple dots indicate Later Jurassic Karabastau Formation of Kazakhstan.

Based on the aforementioned phylogenetic results, we present three outgroups and five families of Evanioidea with their respective geological time in Figure 12. Evanioidea is an important superfamily of Hymenoptera which appeared during the Jurassic radiation. Up to date, more than 35 fossil genera and 110 fossil species of Evanioidea from 18 countries have been published [29]. Evanioidea is an ancient and diverse taxon in Apocrita, however, its origins have not been clearly elucidated. In 2010, Rasnitsyn and Zhang [24] proposed a view of the early evolution of Apocrita: Karatavitidae as ancestral to Orussoidea and to Ephaltitidae, and in turn Ephaltitidae as ancestral to Stephanidae, to Evanioidea, and to (Ceraphronomorpha + Proctotrupomorpha + (Ichneumonomorpha + Vespomorpha)). The results of our current study are consistent with relevant part of their proposal: Karatavitidae appears a sister of Ephaltitidae, Kuafuidae and Evanioidea; the three families are considered to be the primitive groups of Apocrita; and Ephaltitidae is the sister group of Kuafuidae and Evanioidea.

In the Evanioidea clade, the extinct family of Praeaulacidae is at the base of the remaining Evanioidea, which is compatible with the statements of Rasnitsyn [1,2] that Praeaulacidae is the ancestral group of Evanioidea. Praeaulacidae display a series of plesiomorphic characters: medial mesonotal suture well developed; forewing venation moderately complete; and external ovipositor long. In particular, Praeaulacidae along with Anomopterellidae are the only Jurassic Apocrita which have the metasomal attachment high, with all (or at least the most) of the posterior propodeal face closed below the metasomal attachment [30]. Evaniidae and Gasteruptiidae as forming a sister group are consistent with previous results [31], whereas Aulacidae is assigned as their sister, and Anomopterellidae as a sister group of (Aulacidae+ (Evaniidae + Gasteruptiidae)).

Anomopterellidae, an extinct family, is the second family of Evanioidea present in the Middle Jurassic of China. Compared to three other families (Aulacidae, Evaniidae and Gasteruptiidae), which appeared in Early Cretaceous [32-34], we consider that the Anomopterellidae is less advanced than its sister group. Anomopterellidae forms an independent branch as a second basal group in Evanioidea mainly due to some derived characters: 2r‑m lost, cell 2mcu as wide (high) as, or narrower than cell 2+3 rm; hind wing RS and M fused for a distance; pronotum variable in respect to its medial length; and mesonotum variable in respect to presence or absence of medial suture.

Two families of Evanioidea, Praeaulacidae and Anomopterellidae, have been discovered from the Middle Jurassic Jiulongshan Formation of Daohugou deposits [2,3,23], and the Late Jurassic Karabastau Formation of Karatau, South Kazakhstan [4,7] and Shar Teg Beds of SW Mongolia [6]. Based on the information of Table S1 (see Additional file), there are 22 species within 8 genera of Praeaulacidae and 3 species within 2 genera of Anomopterellidae in the Karatau fauna, whereas 25 species within 7 genera of Praeaulacidae and 7 species within 2 genera of Anomopterellidae in the Daohugou fauna [2-4,7,35,36]. These data highlight: (1) the Praeaulacidae and Anomopterellidae are diverse in the Daohugou fauna and Karatau fauna, (2). five common genera in the two faunas, namely *Aulacogastrinus*, *Praeaulacon*, *Praeaulacus* and *Nevania* of Praeaulacidae and *Anomopterella* of Anomopterellidae, indicating a close relationship between the two regions; (3) many genera and species of Praeaulacidae and Anomopterellidae in the late Middle Jurassic of Daohugou suggesting that Evanioidea at least appeared before the late Middle Jurassic.

## Conclusions

Based on a series of plesiomorphic characters, Praeaulacidae has the most basal position in the Evanioidea. Anomopterellidae, as the second family of Evanioidea reported in the Middle Jurassic of China, forms an independent branch and a second most basal group in Evanioidea. Within Anomopterellidae, *Choristopterella* gen. nov. is the most plesiomorphic anomopterellid in respect of RS+M short and not reaching 1m-cu. However, this genus is highly apomorphic in small size and very short propodeum with comparatively low metasomal attachment. Comparing the size of all described anomopterellids, the Daohugou specimens are larger than Kazakhstan specimens, with the only known Mongolian isolated forewing being intermediate in length. Two families of Evanioidea, Praeaulacidae and Anomopterellidae, have a high diversity in the late Middle Jurassic Jiulongshan Formation in Daohugou, suggesting that Evanioidea appeared before the Middle Jurassic.

## Supporting Information

Table S1 **Genera and species of Evanioidea from Jiulongshan Formation and Karabastau Formation.** (M. Jur. —Middle Jurassic; L. Jur. —Later Jurassic.).
(DOC)
