# Supplementary material for: Anomopterellidae Restored, with Two New Genera and Its Phylogeny in Evanioidea (Hymenoptera)
Source: PLoS One. 2013 Dec 10;8(12):e82587. doi: 10.1371/journal.pone.0082587 (PMC3858340; doi:10.1371/journal.pone.0082587)
Supplement: Table S1 — Genera and species of Evanioidea from Jiulongshan Formation and Karabastau Formation. (M. Jur. —Middle Jurassic; L. Jur. —Later Jurassic.). (DOC) [file pone.0082587.s001.doc]

**Table S1.** Genera and species of Evanioidea from Jiulongshan Formation and Karabastau Formation. (M. Jur.—Middle Jurassic; L. Jur.—Later Jurassic.)

| **Taxa** | Occurrence | Reference |
| --- | --- | --- |
| **Praeaulacidae**  1. *Aulacogastrinus ater* Rasnitsyn, 1983  2. *Aulacogastrinus hebeiensis* Zhang & Rasnitsyn, 2008  3. *Aulacogastrinus insculptus* Zhang & Rasnitsyn, 2008  4. *Aulacogastrinus longaciculatus* Zhang & Rasnitsyn, 2008  5. *Aulacogastrinus* sp.  6. *Eosaulacus giganteus* Zhang & Rasnitsyn, 2008  7. *Eosaulacus granulatus* Zhang & Rasnitsyn, 2008  8. *Evaniops rostratus* Rasnitsyn, 1972  9. *Praeaulacinus crassipes* Rasnitsyn, 1972  10. *Praeaulacinus minor* Rasnitsyn, 1972  11. *Praeaulacinus parvus* Rasnitsyn, 1973  12.*Praeaulacites medius* Rasnitsyn, 1972  13. *Praeaulacites minimus* Rasnitsyn, 1972  14. *Praeaulacites nigripes* Rasnitsyn, 1972  15. *Praeaulacites pachygaster* Rasnitsyn, 1972  16. *Praeaulacites subniger* Rasnitsyn, 1972  17. *Praeaulacon caudatus* Rasnitsyn, 1972  18. *Praeaulacon coniventer* Rasnitsyn, 1972  19. *Praeaulacon elongatus* Rasnitsyn, 1972  20. *Praeaulacon tenuis* Rasnitsyn, 1972  21. *Praeaulacon elegantulus* Zhang & Rasnitsyn, 2008  22. *Praeaulacon ningchenggensis* Zhang & Rasnitsyn, 2008  23. *Praeaulacops lucidus* Rasnitsyn, 1972  24. *Praeaulacus afflatus* Zhang & Rasnitsyn, 2008  25. *Praeaulacus cubocephalus* Rasnitsyn, 1972  26. *Praeaulacus douhugouensis* Zhang & Rasnitsyn, 2008  27. *Praeaulacus scabratus* Zhang & Rasnitsyn, 2008  28. *Praeaulacus sculptus* Zhang & Rasnitsyn, 2008  29. *Praeaulacus elegans* Rasnitsyn, 1972  30. *Praeaulacus exquisitus* Zhang & Rasnitsyn, 2008  31. *Praeaulacus leptogaster* Rasnitsyn, 1972  32. *Praeaulacus magnus* Rasnitsyn, 1972  33 *.Praeaulacus obscurus* Rasnitsyn, 1972  34. *Praeaulacus orientalis* Zhang & Rasnitsyn, 2008  35. *Praeaulacus ramosus* Rasnitsyn, 1972  36. *Praeaulacus robustus* Zhang & Rasnitsyn, 2008  37. *Nevania delicata* Zhang & Rasnitsyn, 2007  38. *Nevania ferocula* Zhang & Rasnitsyn, 2007  39. *Nevania malleata* Zhang & Rasnitsyn, 2007  40. *Nevania robusta* Zhang & Rasnitsyn, 2007  41. *Nevania exquisita* Zhang & Rasnitsyn, 2007  42. *Nevania retenta* Zhang & Rasnitsyn, 2007  43. *Nevania karatau* Zhang & Rasnitsyn, 2008  44. *Eonevania robusta* Rasnitsyn & Zhang, 2010  45. *Sinaulacogastrinus eucallus* Zhang & Rasnitsyn, 2008  46. *Sinaulacogastrinus solidus* Rasnitsyn & Zhang, 2010  47. *Sinevania speciosa* Rasnitsyn & Zhang, 2010  **Anomopterellidae**  48. *Synaphopterella patula* Li, Rasnitsyn, Shih & Ren, sp. nov.  49. *Choristopterella stenocera* Li, Rasnitsyn, Shih & Ren,  50. *Anomopterella mirabilis* Rasnitsyn, 1975  51. *Anomopterella huangi* Zhang & Rasnitsyn, 2008  52. *Anomopterella coalita* Li, Rasnitsyn, Shih & Ren, sp. nov.  53. *Anomopterella ampla* Li, Rasnitsyn, Shih & Ren, sp. nov.  54. *Anomopterella brachystelis* Li, Rasnitsyn, Shih & Ren, sp. nov.  55 .*Anomopterella divergens* Li, Rasnitsyn, Shih & Ren, sp. nov*.*  56. *Anomopterella ovalis* Li, Rasnitsyn, Shih & Ren, sp. nov*.* | Kazakhstan; L. Jur.  China; M. Jur.  China; M. Jur.  China; M. Jur.  China; M. Jur.  China; M. Jur.  China; M. Jur.  Kazakhstan; L. Jur.  Kazakhstan; L. Jur.  Kazakhstan; L. Jur.  Kazakhstan; L. Jur.  Kazakhstan; L. Jur.  Kazakhstan; L. Jur.  Kazakhstan; L. Jur.  Kazakhstan; L. Jur.  Kazakhstan; L. Jur.  Kazakhstan; L. Jur.  Kazakhstan; L. Jur.  Kazakhstan; L. Jur.  Kazakhstan; L. Jur.  China; M. Jur.  China; M. Jur.  Kazakhstan; L. Jur.  China; M. Jur.  Kazakhstan; L. Jur.  China; M. Jur.  China; M. Jur.  China; M. Jur.  Kazakhstan; L. Jur.  China; M. Jur.  Kazakhstan; L. Jur.  Kazakhstan; L. Jur.  Kazakhstan; L. Jur.  China; M. Jur.  Kazakhstan; L. Jur.  China; M. Jur.  China; M. Jur.  China; M. Jur.  China; M. Jur.  China; M. Jur.  China; M. Jur.  China; M. Jur.  Kazakhstan; L. Jur.  China; M. Jur.  China; M. Jur.  China; M. Jur.  China; M. Jur.  China; M. Jur.  Kazakhstan; L. Jur.  Kazakhstan; L. Jur.  China; M. Jur.  China; M. Jur.  China; M. Jur.  China; M. Jur.  China; M. Jur.  China; M. Jur. | Rasnitsyn 1972, 1983  Zhang & Rasnitsyn, 2008  Zhang & Rasnitsyn, 2008  Zhang & Rasnitsyn, 2008  Zhang & Rasnitsyn, 2008  Zhang & Rasnitsyn, 2008  Zhang & Rasnitsyn, 2008  Rasnitsyn 1972  Rasnitsyn 1972  Rasnitsyn 1972  Rasnitsyn 1972, 1973  Rasnitsyn 1972  Rasnitsyn 1972  Rasnitsyn 1972  Rasnitsyn 1972  Rasnitsyn 1972  Rasnitsyn 1972  Rasnitsyn 1972  Rasnitsyn 1972  Rasnitsyn 1972  Zhang & Rasnitsyn, 2008  Zhang & Rasnitsyn, 2008  Rasnitsyn 1972  Zhang & Rasnitsyn, 2008  Rasnitsyn 1972  Zhang & Rasnitsyn, 2008  Zhang & Rasnitsyn, 2008  Zhang & Rasnitsyn, 2008  Rasnitsyn 1972  Zhang & Rasnitsyn, 2008  Rasnitsyn 1972  Rasnitsyn 1972  Rasnitsyn 1972  Zhang & Rasnitsyn, 2008  Rasnitsyn 1972  Zhang & Rasnitsyn, 2008  Zhang & Rasnitsyn, 2007  Zhang & Rasnitsyn, 2007  Zhang & Rasnitsyn, 2007  Zhang & Rasnitsyn, 2007  Zhang & Rasnitsyn, 2007  Zhang & Rasnitsyn, 2007  Zhang & Rasnitsyn, 2008  Rasnitsyn & Zhang, 2010  Zhang & Rasnitsyn, 2008  Rasnitsyn & Zhang, 2010  Rasnitsyn & Zhang, 2010  This study  Rasnitsyn, 1975  Rasnitsyn, 1975  Zhang & Rasnitsyn, 2008  This study  This study  This study  This study  This study |
